# Parabolic dependence of the drag coefficient on wind speed from aircraft eddy-covariance measurements over the tropical Eastern Pacific

**DOI:** 10.1038/s41598-020-58699-9

**Published:** 2020-02-04

**Authors:** Zhiqiu Gao, Wenwu Peng, Chloe Y. Gao, Yubin Li

**Affiliations:** 1 Collaborative Innovation Center on Forecast and Evaluation of Meteorological Disasters, School of Atmospheric physics, Nanjing University of Information Science and Technology, Nanjing, 210044 China; 2 State Key Laboratory of Atmospheric Boundary Layer Physics and Atmospheric Chemistry, Institute of Atmospheric Physics, Chinese Academy of Sciences, Beijing, 100029 China; 3 School of Applied Meteorology, Nanjing University of Information Science and Technology, Nanjing, 210044 China; 4 Department of Civil and Environmental Engineering, Massachusetts Institute of Technology, Cambridge, MA 02139 United States; 5 Southern Marine Science and Engineering Guangdong Laboratory (Zhuhai), Zhuhai, 519082 China

**Keywords:** Atmospheric dynamics, Physical oceanography

## Abstract

In this study, we examine and present the relationship between drag coefficient and wind speed. We used an observational dataset that consists of 806 estimates of the mean flow and fluxes from aircraft eddy-covariance measurements over the tropical Eastern Pacific. To estimate the saturated wind speed threshold, we regressed the drag coefficients for wind speed scope from 10 ms^−1^ to 28 ms^−1^. Results show that the relationship between drag coefficient and wind speed is parabolic. Additionally, the saturated wind speed threshold is 22.33 ms^−1^ when regressed from drag coefficient, and it is 22.65 ms^−1^ when regressed from the medium number of drag coefficient for each bin.

## Introduction

The turbulent momentum exchange at the sea surface can be described in terms of drag coefficient (*C*_*d*_) and wind speed. Parameterization of drag coefficient over the air-sea interface is essential to many aspects of air-sea interaction, which is vital for atmospheric, oceanic and surface wave prediction models, as well as climate modeling. Early studies established different linear relationships between drag coefficient and wind speed^1–3^ and dependence relationships of drag coefficient on wind speed and wave status parameters^4–7^ (wave age, wave height, and wave steepness) from field and laboratory observations. However, these studies are mostly only applicable to low-to-moderate wind conditions, and they are unsuitable for high wind conditions due to effects of sea spray droplets produced by bursting bubbles and/or wind tearing breaking wave crests^8^. The drag coefficient under high wind conditions and its parameterization have drawn a growing interest in recent years. Simulating a tropical storm boundary layer by constructing an annular wind wave tank, Alamaro *et al*. concluded that both the drag coefficient and aerodynamic roughness increase with the 10-m wind speed that ranges from 4 ms^−1^ to 35 ms^−1^, and decrease with the 10-m wind speed when it is higher than 35 ms^−1^^9^. Powell *et al*. captured the behavior of the drag coefficient using their Global Positioning System sonde observations in tropical cyclone environments. They found that the drag coefficient would reach its peak when the wind speed is approximately 33 ms^−1^^10^. In their laboratory extreme wind experiments, Donelan *et al*. found that the drag coefficient is 0.0025, and the aerodynamic roughness approaches a limiting value (0.00335 m) under high winds conditions (>33 ms^−1^), while providing a fluid mechanical explanation to their observation^11^. Solving the turbulent kinetic energy balance equation for airflow under the limited saturation (by suspended sea-spray droplets) regime, Makin predicted the reduction of the drag coefficient exceeding hurricane values of 30–40 ms^−1^^12^. Kudryavtsev and Makin extended the wind-over-waves coupling model to high wind speeds by taking into account the sheltering effect of the short wind waves by the air-flow separation from breaking crests of longer waves^13^. At high wind speeds, up to 60 ms^−1^, the modeled aerodynamic roughness is consistent with the Charnock relation. Black *et al*. investigated data collected during the Coupled Boundary Layer Air-Sea Transfer (CBLAST) Experiment. They found that the magnitude of the drag coefficient became nearly constant at wind speeds above the 23 ms^−1^ threshold^14^. This result is 10–12 ms^−1^ less than the hurricane-force threshold of 33 ms^−1^ obtained by the GPS drop sonde measurements^10^ and the laboratory tank measurements^11^. Troitskaya *et al*. calculated theoretically and experimentally the laboratory saturation of the drag coefficient at wind speeds exceeding 25 ms^−1^^15^. Soloviev *et al*. verified the increase of the drag coefficient with wind speed up to 30 ms^−1^ using the unified wave-form and two-phase parameterization model^16^. Golbraikh and Shtemler proposed a semi-empirical model for the estimation of the foam impact on the variation of the drag coefficient^17^. They found that the wind speed, at which the fractional foam coverage is saturated, to be responsible for the difference in the drag coefficient behavior under laboratory and open-ocean conditions. As Donelan pointed out, previous studies explored the physics behind field or laboratory observations, however, they did not provide a simple prescription that may be used in a fully coupled (atmosphere-wave-ocean) hurricane prediction model^18^. Donelan revealed a similar Reynolds number dependence of the oceanic sheltering coefficient, as well as a drag coefficient function of Reynolds number, wave age, and wind speed^18^. They showed that the drag coefficient reached its peak at a wind speed of 30 ms^−1^. However, the equations derived bring more challenges to modeling efforts, due to its constantly changing parameters that cannot be measured easily during high wind events^18^. Green and Zhang proposed an empirical quadratic equation to parameterize the drag coefficient from the 10-m wind speed^19^. Peng and Li proposed a parabolic model of the drag coefficient for storm surge simulations in the South China Sea^20^. There is a clear lack of agreement on the parameterization of the sea surface drag coefficient under high wind conditions in the scientific community^21,22^.

Unlike most of the prior studies, this study is to examine mathematically the dependences of the drag coefficient on wind speed by using the aircraft data collected during the Gulf of Tehuantepec Experiment (GOTEX) on the Pacific coast of the Isthmus of Tehuantepec, Mexico, in February 2004. The main objective of this paper is to develop new parameterization equations of the sea surface drag coefficient (*Cd*) dependent solely on wind speed.

## Materials and Methods

### Database

**Figure 1:**
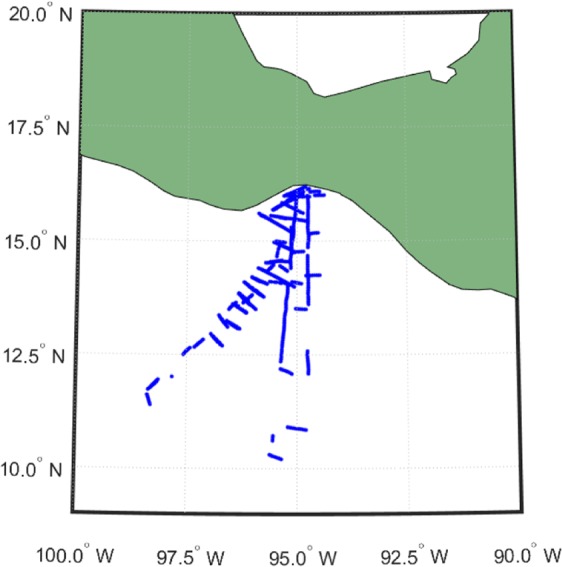
The Geographic locations and flight patterns of the GOTEX experiment.

The turbulent fluxes of momentum, heat, and water vapor used in this study were derived from high-resolution measurements of wind speed, air temperature, and water vapor collected by the National Center for Atmospheric Research (NCAR) C-130 Hercules aircraft in the Gulf of Tehuantepec Experiment (GOTEX) on the Pacific coast of the Isthmus of Tehuantepec, Mexico, in February 2004, where not many studies have been conducted^23,24^ The geographic locations of the aircraft experiments and points (dots) where the data were collected on the flight tracks are shown in Fig. 1. The height of the mixed layer was 500 m and the height of the surface layer was assumed to be around 50–100 m during the experimental period. The wind measurements were obtained close to the surface (between 25 and 50 m *a.s.l*.) from the five-hole gust probe system located on the radome of the aircraft. The fluctuating pressure signals of the five-hole gust probe system were averaged over a period of 5 seconds to allow for conditions to reach steady-state, so the response time is 5 s. The air temperature was determined from one of the Rosemount thermometers with response time of 5 s. and the specific humidity was derived from one of the Lyman-alpha sensors with response time of 0.1 s. Turbulent momentum, heat and water vapor fluxes were obtained as the covariance of the fluctuations from the mean values, averaged over time period of 40 s, which correspond roughly to spatial segments of 4 km at the typical aircraft speed. The mean values were determined over each segment^24^.

### Methods

The sea surface turbulent transfer coefficients for momentum (*Cd*, usually referred as ‘drag coefficient’), heat (*Ch*) and water vapor (*Ce*) are generally defined as

1 $$Cd\equiv \frac{{u}_{\ast }^{2}}{{u}^{2}+{v}^{2}}=\frac{\sqrt{{(\overline{w\text{'}u\text{'}})}^{2}+{(\overline{w\text{'}v\text{'}})}^{2}}}{{U}^{2}},$$

2 $$Ch\equiv \frac{\overline{w\text{'}T\text{'}}}{U({T}_{0}-{T}_{air})},$$

3 $$Ce\equiv \frac{\overline{{w}^{\text{'}}{q}^{\text{'}}}}{U({q}_{0}-{q}_{air})},$$

where u_*_ is the friction velocity, and ${u}_{\ast }\equiv \sqrt{{(\overline{w\text{'}u\text{'}})}^{2}+{(\overline{w\text{'}v\text{'}})}^{2}}$; *u* and *v* are the components of horizontal wind speed in the longitude direction and the latitude direction, respectively; *w* is the vertical wind speed; *u*', *v*' and *w*' are the turbulence fluctuations of *u*, *v* and *w*, respectively; and the overbars indicate the time average; *T*_0_ and *T*_*air*_ are the air temperatures at the sea surface and at the measurement height, and *T*_0_ is considered to be equal to sea surface temperature; *q*_0_ and *q*_*air*_ are air specific humidity at the sea surface and at the measurement height, and *q*_0_ is calculated from the sea surface temperature^25^.

$$U\equiv \sqrt{{u}^{2}+{v}^{2}}.$$

## Results and Discussion

### The variation of friction velocity (u_*_) against wind speed (*U*)

**Figure 2:**
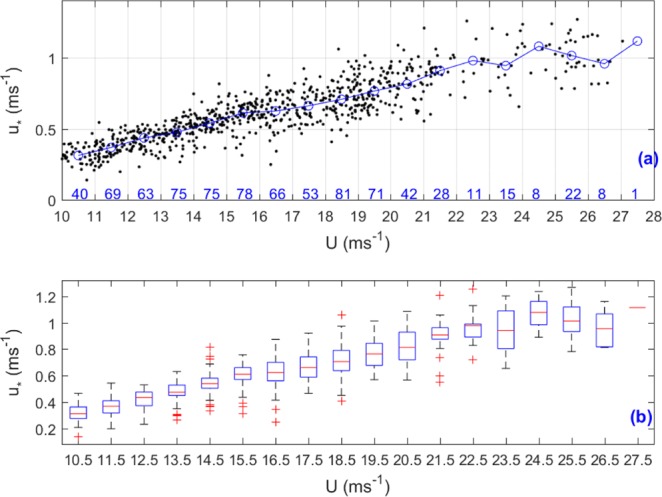
(**a**) The scattered plot of friction velocity (u_*_) against wind speed (*U*) measured during the GOTEX aircraft experiments. The blue line with circle is the median number line and the number of samples for each bin of data was also labeled in blue, and (**b**) the median values (red dashes) and interquartile ranges (blue boxes) of u_*_ for each bin. The red plus symbols are outliners.

Figure 2a shows the scatterplot of friction velocity (u_*_) against wind speed (*U*) collected from the GOTEX experiment. Note that we removed data with wind speeds less than 10 ms^−1^ and use only data collected under high wind conditions as the focus of this study. Overall, u_*_ increased with increasing *U*. The correlation coefficient between u_*_ and *U* is 0.88. The low correlation coefficient between u_*_ and *U* and the discrete distribution of points in Fig. 2a are due to the fact that u_*_ depends not only on *U*, but also on atmospheric stratification stability and sea surface roughness length, which is related to sea surface state (e.g., wave steepness and wave age)^4–6,26^. We classed the data into 18 bins of wind speed at an interval of 1 ms^−1^, and the number of samples for each bin was also labeled in blue in Fig. 2a. The median values (red dashes) and interquartile ranges (blue boxes) of u_*_ for each bin were plotted in Fig. 2b. The red plus symbols are outliners.

### Parameterization of drag coefficient (***Cd***)

**Figure 3:**
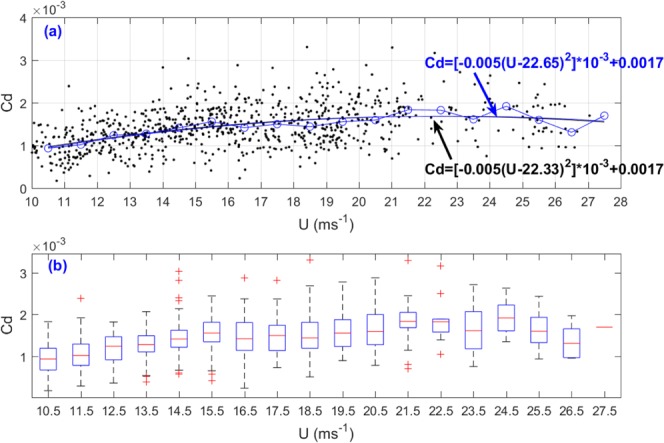
Similar to Fig. 2, but for drag coefficient (*Cd*). (**a**) the blue line with circle is the median number line. The back line is parabolic regression result for all 806 estimates and the blue line is parabolic regression result for the median number for each bin, and (**b**) the median values (red dashes) and interquartile ranges (blue boxes) of *Cd* for each bin. The red plus symbols are outliners.

The drag coefficient (*Cd*) was calculated using Eq. (1). Figure 3a is a scatterplot of drag coefficient (*Cd*) against wind speed (*U*), and the median of these observations for each bin is also shown in blue line with circles. *Cd* increased with increasing *U*. We tried to use polynomial, exponential, Fourier, Gaussian, and linear functions to regress the relationship between *Cd* and *U*. We found that the parabolic relationship obtains the minimum root mean square error (RMSE) and the maximum correlation coefficient, so we regressed the relationship between the drag coefficient *Cd* and *U*:

4 $$C{d}_{}=-\,0.005\times {10}^{-3}{({U}_{}-22.33)}^{2}+0.0017,$$

Applying the regression method for the median numbers of bins, we regressed the relationship between the bin median numbers of *Cd* and *U*:

5 $$C{d}_{}=-\,0.005\times {10}^{-3}{({U}_{}-22.65)}^{2}+0.0017.$$

We find the parabolic relationships between the drag coefficient *Cd* and *U* here. Equations (4) and (5) are very closed to each other. We recommend Eq. (5) because the median method avoids the errors caused by those data points which are too discrete. The “22.65” in Eq. (5) represents the critical (or saturated) wind speed at which *Cd* reaches its maximum value (0.0017). The result of “22.65” obtained here is lower than results from previous studies^9–11^. The possible reason is that the wind speeds used in our work are lower than 28 ms^−1^, and the limited wind speed range brings uncertainty to the regression analysis results. The median values (red dashes) and interquartile ranges (blue boxes) of *Cd* for each bin were plotted in Fig. 3b. The red plus symbols are outliners.

**Figure 4:**
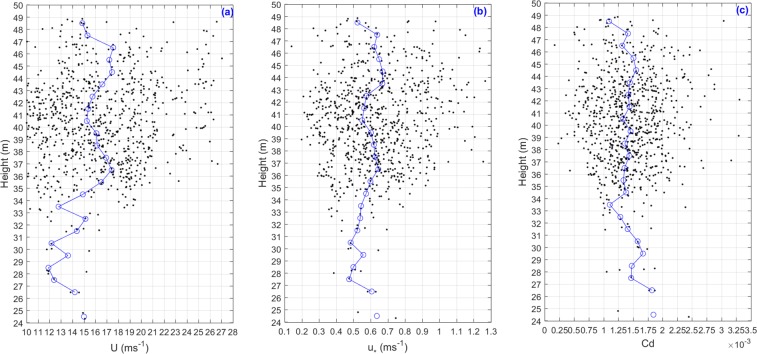
The vertical distribution of (**a**) wind speed (*U*), (**b**) friction velocity (u_*_), and (**c**) drag coefficient (*Cd*). measured during the GOTEX aircraft experiments. The blue line with circle is the median number line.

In this study, we calculated the drag coefficient directly from the wind speed measured by aircrafts, and we did not convert the wind speed measured by the aircrafts to the wind speed at a height of 10 meters, since the logarithmic wind profile hypothesis and the constant flux layer hypothesis over the layer may bring additional errors. Recently, by using the data collected during two Floating Instrument Platform field campaigns and the data collected at the Air-Sea Interaction Tower site, Mahrt *et al*. investigated the relationship between the wind and sea surface stress for contrasting conditions, resulting that the sea surface wind stress decreases significantly with height near the surface under thin marine boundary layers and/or enhanced stress divergence close to the sea surface conditions^27^. We plotted variations of *U*, u_*_ and *Cd* against height in Fig. 4. It is obvious that the most of data collected at the heights range from 31 m and 49 m. Over all, *U* increases slightly and u_*_almost keeps a constant with increasing height, so *Cd* decrease slightly with increasing height.

Equation (5) implies that *Cd* is negative when *U* > 41.09 ms^−1^. Since there is no data higher than 28 ms^−1^ in our study, we carefully constrain the applicable domain of Eq. (5) to between 10 ms^−1^ and 28 m^−1^. Definite conclusions require more extensive measurements under strong wind conditions.

### Parameterizations of turbulent heat transfer coefficient (***Ch***), and turbulent water vapor transfer coefficient (***Ce***)

**Figure 5:**
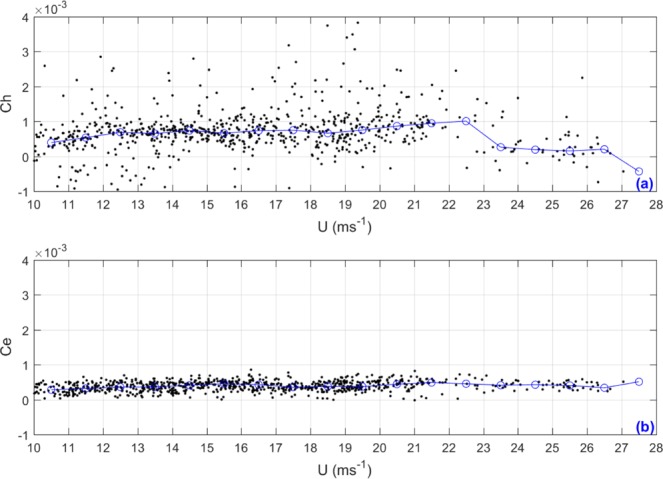
Similar to Fig. 3, but for turbulent heat transfer coefficient (*Ch*) and water vapor transfer coefficient (*Ce*).

In numerical weather forecasting or climate prediction models, parametric drag coefficients, heat transfer coefficients, and water vapor transfer coefficients are usually required at the same time. Do the heat transfer coefficients and water vapor transfer coefficients also have a parabolic increasing behavior with increasing wind speed? Fig. 5 consists two scatterplots of turbulent heat transfer coefficient (*Ch*) and water vapor transfer coefficient (*Ce*) with increasing wind speed (*U*). Figure 5a shows that the distribution of *Ch* is more scattered than *Cd* shown in Fig. 3. The reason is that turbulent heat transfer depends not only on the dynamic process but also on the thermal process, and therefore has more complexity and uncertainty. Figure 5 shows that *Ch* almost remains unchanged when the wind speed is less than 22.65 ms^−1^, suddenly decreases when *U* reaches at 22.65 ms^−1^ and remains at lower values when *U* is higher than 22.65 ms^−1^. This is because when the wind speed is greater than 22.65 ms^−1^, the atmospheric temperature measured by the aircraft remains almost constant (22.42 °C). Unlike Fig. 5a,b shows that the distribution of turbulent water vapor transport coefficients (*Ce*)is relatively concentrated. This is because we assumed that the surface water vapor is saturated during the calculation of *Ce*. The median number lines are also plotted on Fig. 5. It is obvious that neither the heat transfer coefficient nor the water vapor transfer coefficient exhibits a parabolic increase with increasing wind speed.

**Figure 6:**
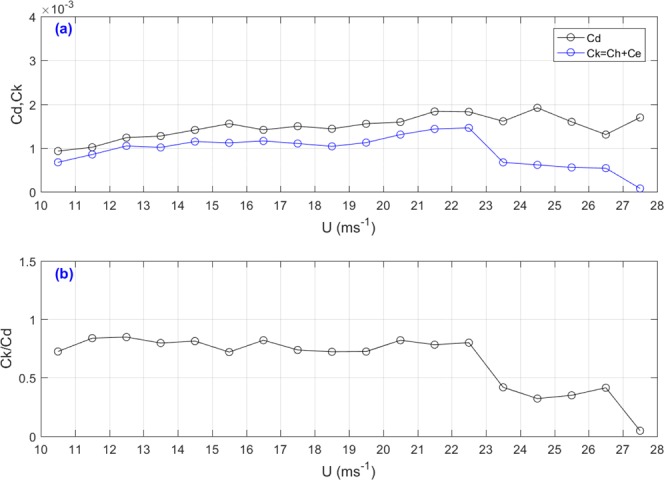
(**a**) The median number variations of drag coefficient (*Cd*) and enthalpy transfer coefficient *Ck* against wind speed (*U*) measured during the GOTEX aircraft experiment; and (**b**) The value of *Ck*/*Cd* against wind speed (*U*) measured during the GOTEX aircraft experiment.

The maximum storm intensity is sensitive to the ratio of the exchange coefficient of enthalpy (*Ck*, the exchange coefficients of heat and water vapor) to the drag coefficient (*Cd*). We plotted enthalpy transfer coefficient *Ck*(≡*Ch* + *Ce*) and *Cd* in Fig. 6a. Alamaro *et al*. deduced that the hurricane intensity depends on the value of *Ck*/*Cd*^9^. Figure 6b shows the variations of *Ck*/*Cd* against wind speed. Figure 6b shows that *Ck* almost remains constant (0.8) when the wind speed is less than 22.65 ms^−1^, suddenly decreases to be 0.4 when *U* reaches at 22.65 ms^−1^ and remain at a lower value (0.4) when *U* is higher than 22.65 ms^−1^. In the previous literature, we rarely see changes in *Ch* with wind speed under strong wind conditions, and we do not see a sudden drop. the value of *Ck*/*Cd*. decreases at *U* = 22.65 ms^−1^, mainly due to the sudden decrease in *Ck*, especially in *Ch*.

## Conclusions

We have established a parabolic relationship between the drag coefficient and wind speed for the data obtained in the GOTEX experiments. By the regression of wind speed and drag coefficient, we found that the saturated wind speed is 22.65 ms^−1^.
